# Modulation of neurosteroid potentiation by protein kinases at synaptic- and extrasynaptic-type GABA_A_ receptors

**DOI:** 10.1016/j.neuropharm.2014.09.021

**Published:** 2015-01

**Authors:** Joanna M. Adams, Philip Thomas, Trevor G. Smart

**Affiliations:** Department of Neuroscience, Physiology & Pharmacology, UCL, Gower Street, London WC1E 6BT, UK

**Keywords:** Neurosteroid, Protein kinase, Synaptic GABA_A_ receptors, Extrasynaptic GABA_A_ receptors, Phosphorylation, THDOC, tetrahydro-deoxycorticosterone, BIM-I, bisindolylmaleimide I, PMA, phorbol-12-myristate-13-acetate, THIP, 4,5,6,7-tetrahydroisothiazolo-[5,4-c]pyridine-3-ol

## Abstract

GABA_A_ receptors are important for inhibition in the CNS where neurosteroids and protein kinases are potent endogenous modulators. Acting individually, these can either enhance or depress receptor function, dependent upon the type of neurosteroid or kinase and the receptor subunit combination. However, *in vivo*, these modulators probably act in concert to fine-tune GABA_A_ receptor activity and thus inhibition, although how this is achieved remains unclear. Therefore, we investigated the relationship between these modulators at synaptic-type α1β3γ2L and extrasynaptic-type α4β3δ GABA_A_ receptors using electrophysiology.

For α1β3γ2L, potentiation of GABA responses by tetrahydro-deoxycorticosterone was reduced after inhibiting protein kinase C, and enhanced following its activation, suggesting this kinase regulates neurosteroid modulation. In comparison, neurosteroid potentiation was reduced at α1β3^S408A,S409A^γ2L receptors, and unaltered by PKC inhibitors or activators, indicating that phosphorylation of β3 subunits is important for regulating neurosteroid activity. To determine whether extrasynaptic-type GABA_A_ receptors were similarly modulated, α4β3δ and α4β3^S408A,S409A^δ receptors were investigated. Neurosteroid potentiation was reduced at both receptors by the kinase inhibitor staurosporine. By contrast, neurosteroid-mediated potentiation at α4^S443A^β3^S408A,S409A^δ receptors was unaffected by protein kinase inhibition, strongly suggesting that phosphorylation of α4 and β3 subunits is required for regulating neurosteroid activity at extrasynaptic receptors. Western blot analyses revealed that neurosteroids increased phosphorylation of β3^S408,S409^ implying that a reciprocal pathway exists for neurosteroids to modulate phosphorylation of GABA_A_ receptors.

Overall, these findings provide important insight into the regulation of GABA_A_ receptors *in vivo*, and into the mechanisms by which GABAergic inhibitory transmission may be simultaneously tuned by two endogenous neuromodulators.

This article is part of the Special Issue entitled ‘GABAergic Signaling in Health and Disease’.

## Highlights

• Neurosteroid potentiation at GABA_A_ receptors is modulated by protein kinase activity.
• Synaptic-type α1β3γ2L receptor potentiation is up or down-regulated by PKC activity.
• PKC-mediated modulation occurs via β3 subunit S408 & S409 phosphorylation.
• Potentiation at extrasynaptic-type α4β3δ receptors is additionally regulated by α4^S443^.
• Neurosteroids facilitate phosphorylation of GABA_A_ receptors via β^S408,S409^.

## 1 Introduction

Neurosteroids and protein kinases are among the most potent modulators of the GABA_A_ receptor, which, when acting individually, can enhance or depress receptor function depending on the nature of the neurosteroid or protein kinase present and to some extent, on the subunit combination of the receptor (Belelli and Lambert, 2005, Moss and Smart, 1996). However, *in vivo*, these agents are unlikely to be temporally discrete modulators at GABA_A_ receptors, and are far more likely to act in concert to achieve high-precision fine-tuning of inhibitory neurotransmission.

A number of previous studies have indicated that the activity of protein kinases, and presumably phosphorylation, can modulate the potentiating effects of selected neurosteroids on both recombinant and native GABA_A_ receptors. For example, in *Xenopus* oocytes expressing recombinant α1β2γ2L GABA_A_ receptors, the potentiation of GABA_A_ receptor-mediated currents by the naturally-occurring neurosteroid, tetrahydro-deoxycorticosterone (THDOC) is enhanced by the activation of protein kinase C (PKC) (Leidenheimer and Chapell, 1997). These results are supported by other studies showing that inhibition of PKC and/or protein kinase A (PKA) resulted in a reduction of neurosteroid sensitivity in neurons from both the hippocampus (Harney et al., 2003) and hypothalamus (Fáncsik et al., 2000). However, whilst many studies conducted to date support a role for protein kinases modulating neurosteroid activity at GABA_A_ receptors, other results are apparently conflicting. Indeed, whilst the aforementioned studies collectively show positive regulation of neurosteroid potentiation by the activity of protein kinases, in both the lamina II neurons of the spinal cord (Vergnano et al., 2007) and the hypothalamic neurons of pregnant rats (Koksma et al., 2003), enhancement of protein kinase activity causes a reduction in the neurosteroid sensitivity of GABA_A_ receptors. This is supported by a more recent study demonstrating that, in the pyramidal neurons of the hippocampus, kindling causes an increase in GABA_A_ receptor phosphorylation which is accompanied by a concomitant decrease in receptor sensitivity to THDOC (Kia et al., 2011).

The reasons underling these discrepancies are currently unclear, but one factor which may affect the relationship between neurosteroids and protein kinase activity is the subunit combination of the GABA_A_ receptor. Although the neurosteroids appear to display only modest changes in potency across most GABA_A_ receptor subtypes (Belelli et al., 2002, Herd et al., 2007), phosphorylation by protein kinases has been shown to differentially alter GABA_A_ receptor function, depending on the receptor isoform (Moss and Smart, 1996), which can even distinguish between different receptor β subunits (McDonald et al., 1998). Therefore, when acting together, it could be envisaged that protein kinases may modulate the activity of neurosteroids at the GABA_A_ receptor in a manner that is dependent upon the receptor isoform. This may explain the variation in previous studies which have utilized different neuronal populations likely to reflect the presence of a mixture of different subsets of GABA_A_ receptors.

In order to examine how protein kinases modulate the activity of neurosteroids in more detail, we investigated the relationship between neurosteroids and protein kinases at GABA_A_ receptors with defined subunit compositions, replicating typical synaptic- and extrasynaptic-type receptor isoforms, by controlling expression in a secondary cell line. In addition, by mutating specific target residues for protein kinases on GABA_A_ receptor subunits, we unveil a mechanism by which protein kinases can reciprocally act to modulate the actions of neurosteroids at these receptors.

## 2 Methods

### 2.1 Molecular biology

cDNAs encoding murine α1, α1^Q241W^, α4, β3, β3^S408A^, β3^S409A^, β3^S408A,S409A^, γ2L, γ2L^S327A,S343A^ and δ GABA_A_ receptor subunits have all been described previously (Moss et al., 1991, Connolly et al., 1996, McDonald et al., 1998, Hosie et al., 2006, Hosie et al., 2009). These cDNA constructs were cloned into the plasmid vector pRK5. The cDNA construct encoding murine α4^S443A^ was generated by site-directed mutagenesis of the wild-type α4 subunit gene using standard PCR methods and the following oligonucleotides: Forward, gccactcgccctgcatttggatctag and reverse, agctgaccccaaagaagctggc, obtained from Eurofins Genomic.

### 2.2 Cell culture and transfection

HEK293 cells were cultured in Dulbecco's modified Eagle medium (DMEM; Gibco) supplemented with 10% v/v foetal calf serum, 2 mM glutamine, 100 U/ml penicillin G, 100 μg/ml streptomycin and incubated at 37 °C in 95% air/5% CO_2_. Cells were transfected using the calcium phosphate precipitation method (using 1 μg of each subunit cDNA and enhanced green fluorescent protein (eGFP) to a total of 4 μg): 20 μl CaCl_2_ (340 mM) plus 24 μl of 2× HBS (280 mM NaCl, 2.8 mM Na_2_HPO_4_, 50 mM HEPES, pH 7.2) per 22 mm coverslip. Cells were used for electrophysiology 24–48 h later. For biochemistry (60 mm culture dishes) cells were transfected with a total of 9 μg of the appropriate cDNA mix.

### 2.3 Electrophysiology

GABA-activated currents were recorded from transfected HEK293 cells continuously perfused with Krebs solution containing: 140 mM NaCl, 4.7 mM KCl, 1.2 mM MgCl_2_, 2.52 mM Glucose, 11 mM HEPES and 5 mM CaCl_2_ (pH 7.4). Whole-cell recordings were performed using patch pipettes (4–5 MΩ) filled with an internal solution (120 mM KCl, 1 mM MgCl_2_, 11 mM EGTA, 10 mM HEPES, 1 mM CaCl_2_ and 4 mM ATP, pH 7.11) in conjunction with an Axopatch 200B amplifier (Axon Instruments). Cells were voltage clamped at −10 mV and currents filtered at 3 kHz (8 pole Bessel filter), digitized (Digidata 1322A, Molecular Devices) and viewed and analysed using Clampex and Clampfit ver 9.2, respectively (Molecular Devices). Cells were continually monitored for access resistance and discarded if this changed by >20%. Drugs were rapidly-applied using a modified U-tube system with 10–90 % response time of 100–150 msec. THDOC was prepared as a 10 mM stock solution in DMSO, and diluted to the appropriate final concentration in Krebs. The effect of the DMSO vehicle alone was negligible. The EC_20_ GABA concentration was pre-determined by constructing GABA concentration–response curves and new curves were generated regularly to adjust for any drift in EC_20_. The EC_20_ GABA currents were recorded at 2 min intervals. The neurosteroid-mediated potentiation was measured by co-application of EC_20_ GABA with 50 nM THDOC, followed by a recovery EC_20_ GABA response. Recording ceased if the responses did not return to baseline GABA-activated levels. Subsequently, cells were perfused with a protein kinase inhibitor or activator diluted in Krebs solution, and applied continuously via the bath. The exceptions to this were the protein kinase G (PKG) inhibitor (KT5823) and protein kinase A (PKA) activator (cAMP), which were applied via the patch electrode. Stocks of staurosporine (1 mM), BIM-I (1 mM), KT5823 (3 mM) and phorbol-12-myristate-13-acetate (PMA; 1 mM) were prepared in DMSO. cAMP (10 mM) was dissolved in distilled water.

### 2.4 Western blotting

Transfected HEK293 cells were treated with 50 nM THDOC or 100 nM PMA as appropriate and were lysed to isolate total protein. Lysis buffer was supplemented with a combination of protease inhibitors (phenylmethyl sulfonyl fluoride and benzamidine) and phosphatase inhibitors (20 mM NaF, 10 mM sodium pyrophosphate and 20 nM calyculin A). Equal amounts of total protein were subjected to Western blotting to assess the expression of GABA_A_ receptor β3 and phosphorylated β3 subunits (on S408 and S409 residues). Total protein was isolated from each transfected cell culture by homogenisation in ice-cold RIPA buffer, followed by cell disruption with repeated freeze–thaw cycles. Equal amounts of total protein were subjected to sodium-dodecyl-sulphate (SDS) polyacrylamide gel electrophoresis and transferred to nitrocellulose membranes. Blotting was performed using 5% w/v milk, or 0.2% BSA, in 0.1% v/v TWEEN-supplemented TRIS-buffered saline (TBS), with blocking for 1 h at room temperature (RT), exposure to primary antibody overnight at 4 °C, and secondary horseradish peroxidase (HRP)-conjugated antibody for 1 h at RT. Following incubation with each antibody, membranes were washed 3× (10 min, RT) with phosphate buffered saline (PBS) and imaged using the ECL detection system (GE Healthcare). Images were quantified using the Western blot plug-in on ImageJ software (Version 1.44p, National Institutes of Health, USA).

### 2.5 Antibodies

The following primary antibodies were used for Western blotting: Rabbit anti-β3-GABA_A_ receptor (1:1000 dilution, Millipore), Rabbit anti-phospho-β3-GABA_A_ receptor (1:1000, phosphorylated S408/409 epitope, gift from Dr J Jovanovic, UCL, UK). A donkey anti-rabbit (1:2500, GE Healthcare) secondary antibody (IgG (H&L)) conjugated to HRP was subsequently used for detection.

### 2.6 Data analysis and statistics

Peak whole-cell GABA-activated currents were analysed using Clampfit ver 9.2. For protein kinase inhibitor or activator experiments, current responses were normalized to the first response elicited by EC_20_ GABA. The potentiation caused by a neurosteroid was measured relative to the preceding response to EC_20_ GABA and expressed as a percentage. All concentration response curves were constructed by plotting mean peak response amplitude against GABA concentration and the data subsequently fitted with the Hill equation:

$$I=1/\left(1+{\left({\text{EC}}_{50}/\left[\text{GABA}\right]\right)}^{n}\right),$$

where I = GABA-activated current, EC_50_ = concentration of GABA inducing 50% of the maximal current response and *n* is the Hill coefficient. Data were graphically represented and analysed using Origin version 6.0 (Microcal). Statistical analyses were undertaken using GraphPad Instat (v.3) employing either a student's *t*-test (two value comparisons) or an ANOVA (three or more value comparisons) followed by an appropriate post-hoc test (as stated in the text) to compare selected data sets.

## 3 Results

To examine the effects of phosphorylation by protein kinases on the potentiation of typical synaptic GABA_A_ receptors by neurosteroids, HEK293 cells were transfected to express α1β3γ2L subunit-containing GABA_A_ receptors. Whole-cell recording was used to assess the magnitude of neurosteroid-mediated potentiation before and after cells were treated with modulators to inhibit or activate protein kinase activity. Peak currents were recorded in response to brief (3 s) applications of either EC_20_ (the concentration eliciting 20% of the maximal GABA response) GABA alone, (baseline control responses), or EC_20_ GABA co-applied with 50 nM of the neurosteroid, THDOC (the potentiated GABA responses). At this concentration neurosteroids act to potentiate GABA_A_ receptor currents. Higher concentrations in the region of 1 μM and above induce proportionately more direct activation of the receptor in the absence of agonist.

## 4 THDOC potentiation at α1β3γ2L GABA_A_ receptors is reduced following protein kinase inhibition

**Fig. 1:**
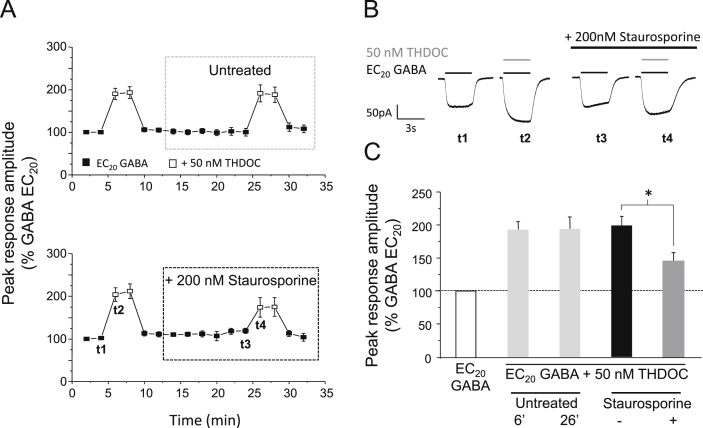
Staurosporine reduces THDOC–mediated potentiation at α1β3γ2L GABA_A_ receptors. (A) Mean peak currents recorded from HEK293 cells expressing α1β3γ2L GABA_A_ receptors in response to EC_20_ GABA (filled squares) or EC_20_ GABA + 50 nM THDOC (open squares). Cells either remained untreated (upper panel) or were exposed to 200 nM staurosporine (lower panel). All responses were normalised to the peak current of the first EC_20_ GABA-activated response, recorded 2 min after achieving the whole-cell recording configuration, designated as *t* = 0 (100%). (B) Example GABA currents taken at the time points shown as t1–t4 in (A, lower panel) alone, or in the presence of 50 nM THDOC (grey bar), before and after staurosporine treatment (black bar). (C) Bar chart showing the potentiation of EC_20_ GABA-activated currents by 50 nM THDOC in untreated cells (light grey bars, measured at 6 and 26 min, respectively; *n* = 6), or in treated cells before (black bar) and after (grey bar) 200 nM staurosporine (*n* = 6). All data points represent mean ± s.e.m. Significant results are indicated by * (*P* < 0.05, paired *t*-test).

To investigate whether neurosteroid-mediated potentiation could be modulated by protein kinases, cells were treated with staurosporine (200 nM), selected as it is broad-spectrum non-specific protein kinase inhibitor. Staurosporine was also chosen because it is cell permeable, which allows the potentiation induced by THDOC to be measured before and after treatment within the same cell, thus reducing any potential variation in results that could arise from using separate populations of treated and untreated cells. For each cell, the baseline response to EC_20_ GABA alone and the potentiated response after co-application of EC_20_ GABA and 50 nM THDOC was established under basal conditions (i.e., before protein kinase inhibitor treatment; Fig. 1A). Cells were then treated with 200 nM staurosporine, applied continuously via the bath perfusion. After 14 min, the baseline and potentiated responses were re-measured. To allow a direct comparison of the potentiation before and after staurosporine treatment, each potentiated response was normalised to the cell's preceding response to EC_20_ GABA alone. For control experiments, cells remained untreated for the duration of the recording and these showed negligible change in the potentiation elicited by 50 nM THDOC over 30 min (2.5 ± 7.9%; *n* = 6; Fig. 1A and C). By contrast, cells treated with staurosporine exhibited a significant decrease in THDOC-mediated potentiation of 54.8 ± 9.1% (*P* < 0.05, paired *t*-test; *n* = 5; Fig. 1A and C). This decrease in potentiation was not caused by a staurosporine-mediated shift in the GABA concentration–response relationship, which remained unchanged (data not shown). Notably, the potentiation by THDOC was not completely abolished, (45.5 ± 12.7% remained after staurosporine treatment: Fig. 1A–C), indicating that whilst THDOC-mediated potentiation of recombinant α1β3γ2L GABA_A_ receptors is modulated by protein kinases, the presence of activated protein kinases *per se*, is not an absolute requirement for neurosteroid potentiation.

## 5 THDOC-mediated potentiation is modulated by PKC

**Fig. 2:**
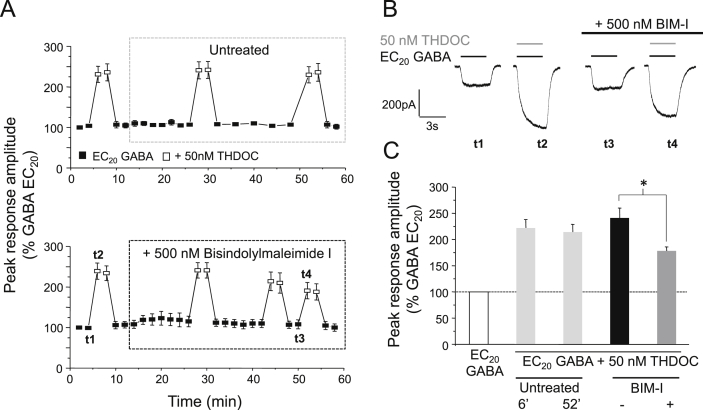
PKC activity modulates THDOC–mediated potentiation at α1β3γ2L GABA_A_ receptors. (A) Mean peak currents recorded from HEK293 cells expressing α1β3γ2L GABA_A_ receptors in response to EC_20_ GABA or EC_20_ GABA + 50 nM THDOC. Cells either remained untreated or were treated with 500 nM bisindolylmaleimide-I (BIM-I). Responses were normalised to the first EC_20_ GABA-activated peak response (*t* = 0 (100%)). (B) Sample whole-cell currents in the presence and absence of 50 nM THDOC (grey bar) before and after BIM-I treatment (black bar). Representative currents are shown from the time points (*t*) indicated in (A: lower panel). (C) Bar chart for the potentiation of EC_20_ GABA-activated currents by 50 nM THDOC in untreated cells (light grey bars, measured at 6 and 52 min, respectively; *n* = 6), or in treated cells before (black bar) and after (grey bar) 500 nM BIM-I treatment (*n* = 5). *P < 0.05, paired *t*-test.

**Supplementary Fig. 1:**
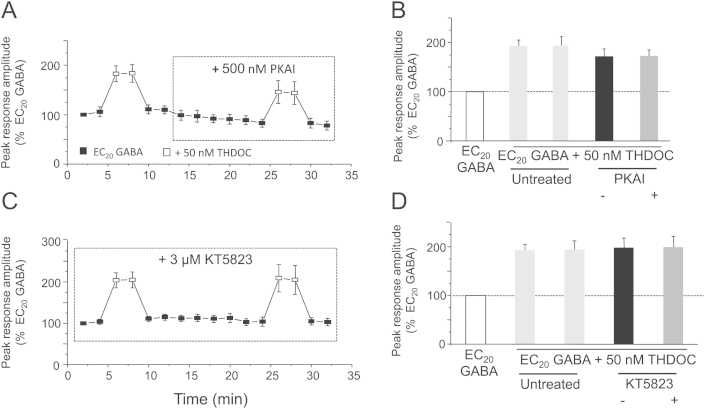
Inhibition of PKA or PKG does not affect THDOC–mediated potentiation at α1β3γ2L GABA_A_ receptors. (A & C) Mean peak currents recorded for α1β3γ2L GABA_A_ receptors in response to EC_20_ GABA or EC_20_ GABA + 50 nM THDOC. Cells were either untreated or exposed to 500 nM myristoylated PKA inhibitor peptide 14-22 amide (PKAI; A) or 3 μM KT5823 (C). (B & D) Bar charts showing the potentiation of EC_20_ GABA-activated currents by 50 nM THDOC in untreated cells (light grey bars; *n* = 6) or in treated cells before (black bars) and after (grey bars) 500 nM PKAI (B; *n* = 5) or 3 μM KT5823 (D; *n* = 6) treatment. *P < 0.05, paired *t*-test.

The GABA_A_ receptor is a target for phosphorylation by a number of serine–threonine protein kinases, including PKA, PKC, PKG and CaMKII (Moss and Smart, 1996, Brandon et al., 2002). To identify the roles that specific protein kinases play in modulating the potentiation of the GABA_A_ receptor by neurosteroids, we treated cells with selective kinase inhibitors or activators. PKC activity was inhibited using 500 nM bisindolylmaleimide I (BIM-I: Fig. 2). Cells treated with BIM-I (for 40 min) exhibited a significant decrease in THDOC-mediated potentiation (by 43.5 ± 3.8%, *P* < 0.05, paired *t*-test; *n* = 5; Fig. 2A and C). By contrast, inhibition of PKA with an inhibitor peptide (500 nM PKAI), or PKG with the selective inhibitor KT5823 (3 μM), did not alter neurosteroid potentiation (Supplementary Fig. 1) indicating that potentiation of α1β3γ2L GABA_A_ receptors by THDOC is modulated primarily by PKC. As observed with staurosporine, the potentiation was not abolished after BIM-I treatment (Fig. 2), further suggesting that protein kinases are not required for basal potentiation by neurosteroids.

## 6 Activation of PKC enhances THDOC-mediated potentiation

**Fig. 3:**
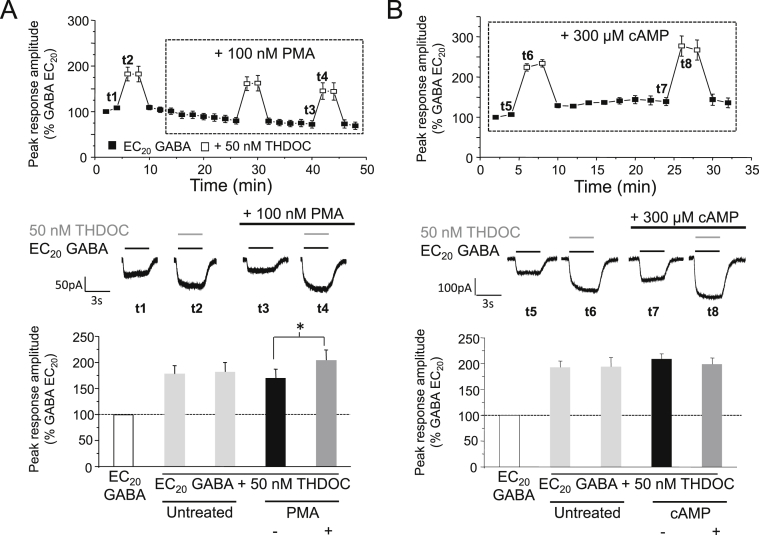
PKC but not PKA increases THDOC–mediated potentiation of α1β3γ2L GABA_A_ receptor currents. (A & B), Top panels: mean peak currents recorded for α1β3γ2L GABA_A_ receptors expressed in HEK cells in response to EC_20_ GABA or EC_20_ GABA + 50 nM THDOC. Cells were untreated or were treated with 100 nM PMA (A) or 300 μM cAMP (B). All currents are normalised as in Fig. 1. Middle panels: typical GABA currents showing potentiation by 50 nM THDOC (grey bar) before and after PMA (A) or cAMP (B). Currents are taken at the respective time points (t1-t4, t5-t8). Lower panels: bar charts for the potentiation of EC_20_ GABA-activated currents by 50 nM THDOC in untreated cells (light grey bars, *n* = 4–6) or in treated cells before (black bars) and after (grey bars) 100 nM PMA (A; *n* = 6) or 300 μM cAMP (B; *n* = 4) treatment. *P < 0.05, paired *t*-test.

To further examine the relationship between PKC and neurosteroid modulation of GABA_A_ receptors, the effects of activating PKC were investigated. For this we used phorbol-12-myristate-13-acetate (PMA). Cells treated with 100 nM PMA (for 36 min) resulted in a significant increase in THDOC-mediated potentiation (by 58.0 ± 18.3%; *P* < 0.05, paired *t*-test; *n* = 6; Fig. 3A). This increase was not due to a PMA induced shift in the GABA concentration–response relationship which remained unchanged (data not shown). We also investigated the effects of activating PKA using cAMP (300 μM) but, contrastingly, this did not significantly alter neurosteroid potentiation (−9.0 ± 4.4%, *P* > 0.05; *n* = 4; Fig. 3B). These observations support those which used the broad-spectrum protein kinase inhibitor staurosporine, and selective inhibitor BIM-I, and indicate that THDOC-mediated potentiation of α1β3γ2L GABA_A_ receptors is primarily modulated by PKC.

**Fig. 4:**
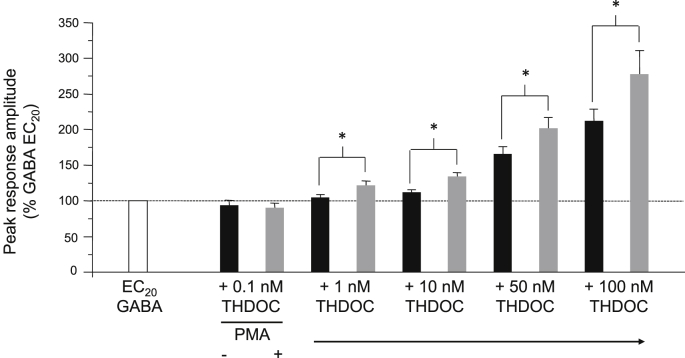
Effect of PMA on the potentiation of α1β3γ2L GABA_A_ receptor currents by varying concentrations of THDOC. Bar chart for the mean potentiations of EC_20_ GABA-activated responses elicited by THDOC (0.1–100 nM) before (black bars) and after (grey bars) 100 nM PMA (*n* = 5–11). All data points represent mean ± s.e.m. *P < 0.05, paired *t*-test.

The concentration of neurosteroids present *in vivo* is not static but instead undergoes dynamic changes in response to various physiological and pathophysiological conditions including stress, pregnancy, the ovarian cycle and ageing (Purdy et al., 1991, Paul and Purdy, 1992, Bäckström et al., 2003, Schumacher et al., 2003, Maguire and Mody, 2009, Sanna et al., 2009), as well as in response to administration of selected psychoactive drugs, such as ethanol, γ-hydroxybutyrate (GHB) and anti-depressants including fluoxetine (Uzunov et al., 1996, Sanna et al., 2004). With this in mind, we performed similar experiments using varying concentrations of THDOC (0.1–100 nM) in order to establish whether PKC can reciprocally modulate the actions of neurosteroids across a range of concentrations. Consistent with previous data, THDOC concentrations of 10 nM and above were able to significantly potentiate α1β3γ2L GABA_A_ receptor function (% potentiation = 12.0 ± 3.5 (10 nM), 66.2 ± 10.0 (50 nM), 111.5 ± 17.2 (100 nM); *P* < 0.05, paired *t*-test; *n* = 5–11; Fig. 4). No significant potentiation was observed in cells exposed to 1 nM or less THDOC (5.3 ± 4.3%, *P* > 0.05; *n* = 8; Fig. 4). After treatment with 100 nM PMA (for 30 min), potentiation by 1, 10, 50 and 100 nM THDOC was significantly enhanced by 102.3 ± 43.2%, 152.7 ± 65.4%, 50.8 ± 14.2% and 34.8 ± 14.2%, respectively (*P* < 0.05, paired *t*-test; *n* = 5–11; Fig. 4). It therefore seems that phosphorylation by PKC can modulate THDOC-mediated potentiation across a broad physiological range of neurosteroid concentrations likely to be experienced *in vivo*. Interestingly, treatment with PMA revealed that potentiation could be elicited by just 1 nM THDOC under conditions of increased PKC activity, which previously was subthreshold for potentiation (Fig. 4).

## 7 Phosphorylation at the β3 subunit is important for modulating THDOC-mediated potentiation

**Fig. 5:**
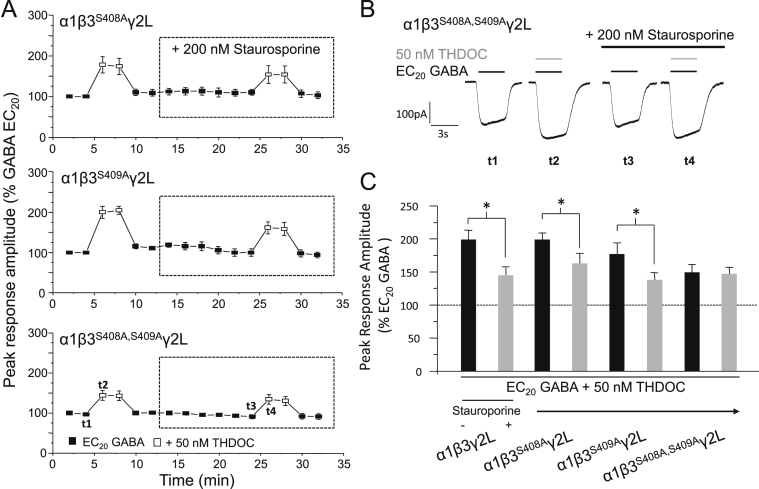
Residues β3^S408A,S409A^ mediate the staurosporine–induced reduction in THDOC potentiation at α1β3γ2L GABA_A_ receptors. (A) Mean peak GABA currents for α1β3^S408A^γ2L (top panel), α1β3^S409A^γ2L (middle panel) and α1β3^S408A,S409A^γ2L (lower panel; all *n* = 5) GABA_A_ receptors in response to EC_20_ GABA or EC_20_ GABA + 50 nM THDOC. Cells were treated with 200 nM staurosporine. All responses were normalised as in Fig. 1. (B) whole-cell currents showing potentiation by 50 nM THDOC (grey bars) before and after staurosporine treatment (black bar) at α1β3^S408A,S409A^γ2L GABA_A_ receptors. (C) Bar chart of the potentiation of EC_20_ GABA-activated currents by 50 nM THDOC before (black bars) and after (grey bars) staurosporine treatment for α1β3γ2L (*n* = 6), α1β3^S408A^γ2L (*n* = 5), α1β3^S409A^γ2L (*n* = 5) and α1β3^S408A,S409A^γ2L (*n* = 5) GABA_A_ receptors. Data for α1β3γ2L is taken from Fig. 1. *P < 0.05, paired *t*-test.

**Supplementary Fig. 2:**
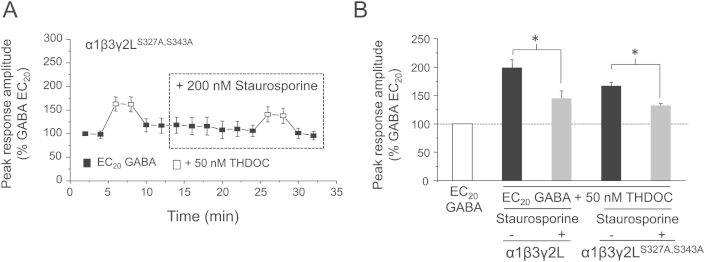
Staurosporine reduces THDOC potentiation at α1β3γ2L^S327A,S343A^ GABA_A_ receptors. (A) Mean peak currents recorded from HEK293 cells expressing α1β3γ2L^S327A,S343A^ (*n* = 4) GABA_A_ receptors in response to EC_20_ GABA or EC_20_ GABA + 50 nM THDOC. Cells were treated with 200 nM staurosporine. (B) Bar chart showing the potentiation of EC_20_ GABA-activated currents by 50 nM THDOC before (black bars) and after (grey bars) staurosporine for α1β3γ2L (*n* = 6) or α1β3γ2L^S327A,S343A^ (*n* = 4) GABA_A_ receptors. Data for α1β3γ2L GABA_A_ receptors is taken from Fig. 1. *P < 0.05, paired *t*-test.

To explore the mechanism by which PKC modulates the activity of neurosteroids at GABA_A_ receptors and, in particular, to examine the involvement of direct receptor phosphorylation in this process, HEK293 cells were transfected to express α1β3γ2L GABA_A_ receptors containing single or multiple point mutations of consensus phosphorylation sites known to be targeted by PKC, namely S408 and S409 on β3 subunits, and S327 and S343, on γ2L subunits. Similar to the results obtained for wild-type receptors, THDOC-mediated potentiation at α1β3^S408A^γ2L, α1β3^S409A^γ2L (Fig. 5A–C) and α1β3γ2L^S327A,S343A^ (Supplementary Fig. 2) GABA_A_ receptors was significantly reduced after treatment (14 min) with 200 nM staurosporine (decrease in potentiation = 36 ± 7.5, 54 ± 6.9% and 50.3 ± 7.5%, respectively, *P* < 0.05, paired *t*-test; *n* = 4–5). By contrast, cells expressing α1β3^S408A,S409A^γ2L GABA_A_ receptors showed no change in the magnitude of the potentiation elicited (−1.74 ± 8.7%, *P* > 0.05; *n* = 5; Fig. 5A–C). This complete removal of sensitivity to kinase inhibition implies that phosphorylation at the β3, but not the γ2L subunit, is important for the PKC-mediated modulation of neurosteroid potentiation at α1β3γ2L GABA_A_ receptors. Furthermore, the requirement to remove both S408 and S409 in order to abolish the staurosporine-induced decrease in THDOC-mediated potentiation, suggests that phosphorylation at either β3 S408 or S409 is sufficient to modulate the actions of neurosteroids at these receptors.

Interestingly, under basal cell conditions (i.e., before staurosporine treatment), the magnitude of potentiation elicited by 50 nM THDOC at α1β3^S408A,S409A^γ2L GABA_A_ receptors was significantly reduced compared to that induced at wild-type receptors (48.6 ± 10.8% versus 99.1 ± 13.8%, *P* < 0.05, ANOVA with Tukey's post hoc test; *n* = 5–6; Fig. 5A, C). This decrease may be due to the β3 subunit (wild-type or either of the single mutants) being basally phosphorylated, a post-translational modification that cannot occur at the doubly mutated subunit. Therefore, as the magnitude of potentiation was reduced under conditions where this subunit cannot be phosphorylated, this supports the notion that phosphorylation at the β3 subunit is important for regulating neurosteroid activity at α1β3γ2L GABA_A_ receptors.

## 8 Phosphorylation by PKC at β3 S408 or S409 is sufficient to modulate neurosteroid activity

**Fig. 6:**
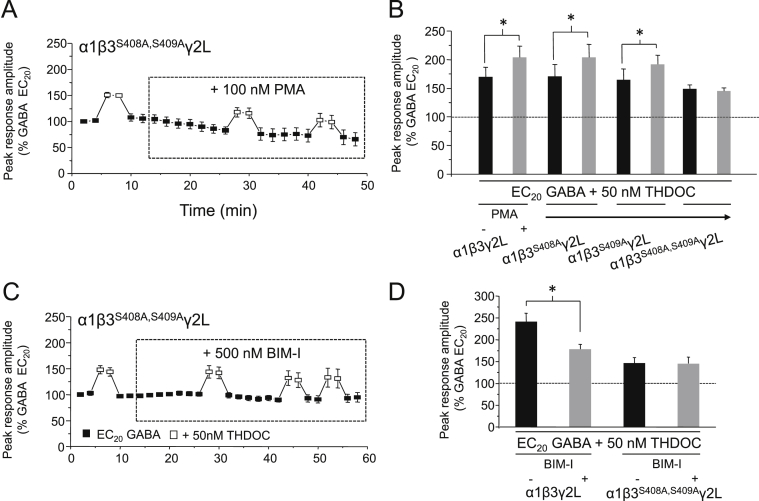
Modulation of PKC activity does not alter neurosteroid potentiation at α1β3^S408A,S409A^γ2L GABA_A_ receptors. (A & C) Mean peak GABA currents for α1β3^S408A,S409A^γ2L (*n* = 4) GABA_A_ receptors in response to EC_20_ GABA or EC_20_ GABA + 50 nM THDOC. Cells were treated with either 100 nM PMA (A) or 500 nM Bisindolylmaleimide I (BIM-I; C). (B & D) Bar charts for the potentiation of EC_20_ GABA-activated currents by 50 nM THDOC before (black bars) and after (grey bars): PMA (B) in cells expressing α1β3γ2L (*n* = 6), α1β3^S408A^γ2L (*n* = 4), α1β3^S409A^γ2L (*n* = 4) or α1β3^S408A,S409A^γ2L (*n* = 5); or BIM-I (D) in cells expressing α1β3γ2L (*n* = 5) or α1β3^S408A,S409A^γ2L (*n* = 4) GABA_A_ receptors. Data for α1β3γ2L is taken from Fig. 2, Fig. 3A. *P < 0.05, paired *t*-test.

We have shown that THDOC-mediated potentiation at α1β3γ2L GABA_A_ receptors is specifically modulated by PKC (Fig. 2). We therefore investigated whether the β3 subunit S408 and S409 residues are targeted specifically by PKC by using either 100 nM PMA or 500 nM BIM-I, in order to specifically activate or inhibit PKC, respectively. Following treatment with 100 nM PMA, cells expressing α1β3^S408A^γ2L or α1β3^S409A^γ2L GABA_A_ receptors exhibited significant enhancements in THDOC-mediated potentiation (54.6 ± 13.8% and 61.1 ± 37%, respectively: *P* < 0.05, paired *t*-test; *n* = 4; Fig. 6B), similar in magnitude to the increase observed at wild-type receptors. By contrast, potentiation at α1β3^S408A,S409A^γ2L receptors was unaffected by treatment with either PMA (Fig. 6A and B: −7.3 ± 9.0%; *P* > 0.05; *n* = 4–5) or BIM-I (Fig. 6C and D; 1.6 ± 8.3%; , *P* > 0.05; *n* = 4–5), indicating that these residues are important for the modulatory effects of PKC observed previously. These results support those obtained using staurosproine and BIM-I, demonstrating that PKC-mediated phosphorylation specifically at β3 S408 and S409 is sufficient to modulate the magnitude of potentiation elicited by 50 nM THDOC at α1β3γ2L GABA_A_ receptors.

The PKC inhibition experiments (Fig. 6C and D) also show that, under basal cell conditions, the magnitude of THDOC-mediated potentiation induced at α1β3^S408A,S409A^γ2L GABA_A_ receptors is significantly reduced compared to that observed at wild-type receptors (45.7 ± 12.9% compared to 140.6 ± 19.2%, respectively, *P* < 0.05, paired *t*-test; *n* = 4–5). This supports the idea that basal phosphorylation at the wild-type receptor, which is eliminated from the double mutant receptor, results in an apparent enhancement in the level of potentiation elicited by 50 nM THDOC. In support of this, it is notable that in the PKC activation experiment, there was a trend for the THDOC (50 nM) potentiation elicited at wild-type receptors (70.2 ± 17.3% enhancement of the EC_20_ GABA-activated current) to be greater than that at α1β3^S408A,S409A^γ2L GABA_A_ receptors (48.8 ± 6.5%; Fig. 6B), though this did not reach significance.

## 9 Basal phosphorylation is not required for THDOC-mediated potentiation

To further explore the requirement for protein kinase activation in the modulation of GABA_A_ receptors by neurosteroids, the magnitude of potentiation elicited by 50 nM THDOC was assessed in cells expressing α1β3^S408A,S409A^γ2L^S327A,S343A^ GABA_A_ receptors, effectively eliminating basal PKC-induced phosphorylation at all known PKC sites within the receptor. Consistent with the protein kinase inhibition experiments, potentiation was still observed at these receptors (data not shown), indicating that phosphorylation by protein kinases is not required for the potentiation of GABA_A_ receptor function by neurosteroids.

## 10 THDOC enhances β3 subunit phosphorylation via an interaction with the GABA_A_ receptor

**Fig. 7:**
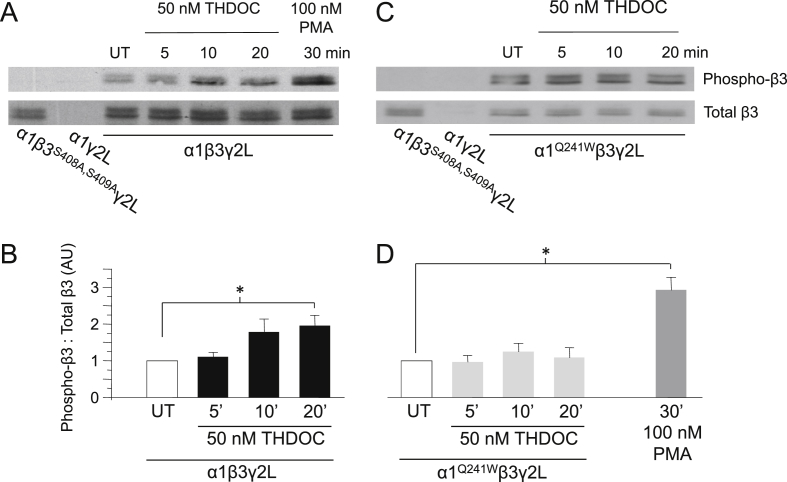
THDOC enhances phosphorylation of β3^S408,^^S409^ by binding to the GABA_A_ receptor. (A,C) Representative Western blots showing the level of β3 subunit phosphorylation in HEK293 cells expressing (A) α1β3γ2L or (C) α1^Q241W^β3γ2L GABA_A_ receptors. Cells were either untreated (UT) or exposed to 50 nM THDOC for 5, 10 or 20 min or 100 nM PMA for 30 min (as indicated) and probed with β3 or phospho-β3 anti-sera. Cells transfected with α1β3^S408A,S409A^γ2L or α1γ2L GABA_A_ receptor subunits were used as controls. (B, D) Bar charts showing average levels of phosphorylated β3 subunits in cells expressing α1β3γ2L (B: black bars; *n* = 3) or α1^Q241W^β3γ2L (D: grey bars; *n* = 3), in UT cells or after 50 nM THDOC for 5, 10 or 20 min, or 100 nM PMA (D) for 30 min. *P < 0.05, ANOVA with Dunnett's post hoc test.

The experiments conducted throughout this study have focused on the ability of phosphorylation, by protein kinases, to modulate the actions of neurosteroids at GABA_A_ receptors. However, this reflects just one aspect of this relationship and does not address the potential for this modulation to be bi-directional, i.e., can neurosteroids affect the extent of phosphorylation of the GABA_A_ receptor? To investigate this, Western blot analysis was used to determine the extent of β3 subunit phosphorylation in HEK293 cells expressing α1β3γ2L GABA_A_ receptors, before and after exposure to a neurosteroid. Cells either remained untreated or were exposed to 50 nM THDOC for 5, 10 or 20 min. Lysates were probed in duplicate with both β3 and phospho-β3 anti-sera and, for each treatment group, the level of phosphorylated β3 was normalized to the corresponding amount of total β3 present. As expected, no phosphorylated β3 was detected in cells expressing α1β3^S408A,S409A^γ2L (Fig. 7A lane 1) and both β3 and phosphorylated β3 were predictably not detected in cells expressing just α1 and γ2L subunits (Fig. 7A lane 2), confirming the specificity of antibodies for their respective targets.

Experiments performed on untreated cells confirmed that there is some basal phosphorylation of the β3 subunit when it is co-expressed with α1 and γ2L in HEK293 cells (Fig. 7A lane 3). After treatment of cells with 50 nM THDOC for 5 min, there was little change in the level of β3 subunit phosphorylation (10.7 ± 11.6% increase, *P* > 0.05; *n* = 3; Fig. 7A lane 4, Fig. 7B). A tendency towards increased phosphorylation was detected in cells treated with THDOC for 10 min (78.7 ± 34.9%, *P* > 0.05; *n* = 3; Fig. 7A lane 5, Fig. 7B), and cells exposed to THDOC for 20 min exhibited a significant enhancement in the extent of β3 subunit phosphorylation of 96.3 ± 27.9% (*P* < 0.05, ANOVA with Dunnett's post hoc test; *n* = 3; Fig. 7A lane 6, Fig. 7B). Of interest, this level of phosphorylation was still significantly less than that achieved by combining a slightly longer incubation time of THDOC (30 min) with 100 nM PMA (Fig. 7A, lane 7). Collectively, these results indicate that THDOC can enhance β3 subunit phosphorylation, supporting the presence of a reciprocal transduction pathway in which neurosteroids can themselves modulate the phosphorylation of the α1β3γ2L GABA_A_ receptor. This in turn may modify the ability of specific kinases to further potentiate the receptor.

In order to examine whether THDOC acts to enhance receptor phosphorylation through an interaction with the GABA_A_ receptor, we repeated our experiments using cells expressing α1^Q241W^β3γ2L GABA_A_ receptors. We previously determined that this mutation is critically responsible for the sensitivity of GABA_A_ receptors to potentiating concentrations of neurosteroid (Hosie et al., 2006). In untreated cells, basal phosphorylation was still evident at the β3 subunit (Fig. 7C lane 3), indicating that these receptors can be normally phosphorylated. However, following exposure to 50 nM THDOC for 5, 10 or 20 min, no significant alteration in the level of β3 subunit phosphorylation was observed (Changes: -2.7 ± 18.4%, 25.0 ± 22.7% and 9.3 ± 27.4%, respectively, *P* > 0.05; *n* = 3; Fig. 7C lanes 4–6, Fig 7D). Notably, 100 nM PMA (30 min) was still able to significantly phosphorylate the α1^Q241W^β3γ2L receptor (Fig. 7D). These results suggest that THDOC acts to modulate GABA_A_ receptor phosphorylation through a direct interaction with the receptor complex. Furthermore, as the mutation Q241W is sufficient to eliminate any enhancement in receptor phosphorylation by neurosteroid, it is apparent that occupancy of the potentiating neurosteroid binding site is necessary to facilitate this reciprocal modulation.

Collectively, these results show that a complex, bi-directional relationship exists between neurosteroids and protein kinases at the α1β3γ2L GABA_A_ receptor, with phosphorylation at the receptor acting to positively modulate the actions of neurosteroids and, in the reverse direction, exposure to neurosteroids enhancing the extent of receptor phosphorylation.

## 11 THDOC potentiation at α4β3δ GABA_A_ receptors is reduced by protein kinase inhibition

In order to investigate whether the activity of protein kinases is also important for regulating the magnitude of neurosteroid potentiation at extrasynaptic-type receptors, HEK293 cells were transfected to express α4β3δ subunit-containing GABA_A_ receptors. To ensure that the expressed receptors contained the δ subunit, sensitivity to the agonist, 4,5,6,7-tetrahydroisothiazolo-[5,4-c]pyridine-3-ol (THIP) was examined. THIP has been shown to exhibit super-agonist activity at δ subunit-containing receptors compared to its partial agonism at αβγ receptors (Brown et al., 2002, Storustovu and Ebert, 2006, Mortensen et al., 2010). We therefore used this agonist as a reliable indicator for the presence of δ subunits within the receptor complex. The results showed that 100 μM THIP-induced currents were significantly larger than those elicited by 100 μM GABA, indicating incorporation of the δ subunit (data not shown).

**Fig. 8:**
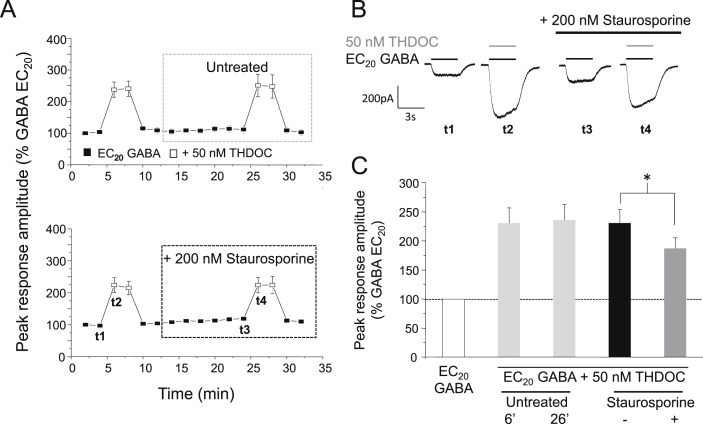
Staurosporine reduces THDOC potentiation at α4β3δ GABA_A_ receptors. (A) Mean peak currents for α4β3δ GABA_A_ receptors activated by EC_20_ GABA or EC_20_ GABA + 50 nM THDOC. Cells were either untreated (upper panel) or exposed to 200 nM staurosporine (lower panel). (B) Sample whole-cell currents in the absence and presence of 50 nM THDOC (grey bar) before and after 200 nM staurosporine treatment (black bar). (C) Bar chart showing the potentiation of EC_20_ GABA-activated currents by 50 nM THDOC in untreated cells (light grey bars, measured at 6 and 26 min, respectively; *n* = 5) or in treated cells before (black bar) and after (grey bar) 200 nM staurosporine treatment (*n* = 9). Responses were normalised to the peak current recorded at 2 min. *P < 0.05, paired *t*-test.

To determine whether the actions of neurosteroids at α4β3δ GABA_A_ receptors could be modulated by the activity of protein kinases, cells were treated with the broad-spectrum protein kinase inhibitor, staurosproine (200 nM) for 14 min. Staurosporine significantly reduced the potentiation induced by 50 nM THDOC (decrease: 38.1 ± 5.8%, *P* < 0.05, paired *t*-test; *n* = 9; Fig. 8A–C). We deduce that the actions of neurosteroids at α4β3δ GABA_A_ receptors can therefore also be modulated by the activity of protein kinases. Furthermore, as observed with α1β3γ2L GABA_A_ receptors, protein kinases are not required for the induction of THDOC-mediated potentiation at these receptors, as 50 nM THDOC remained able to enhance the EC_20_ GABA-activated current by 83.7 ± 18.5% after staurosporine treatment (Fig. 8A–C).

A number of studies have reported that GABA_A_ receptors incorporating the δ subunit are more sensitive to the potentiating actions of neurosteroids (Belelli et al., 2002, Brown et al., 2002, Wohlfarth et al., 2002). However, in the present study, the magnitude of potentiation elicited by 50 nM THDOC at α4β3δ GABA_A_ receptors did not differ significantly from that observed at α1β3γ2L receptors (128 ± 23.6% and 99.1 ± 13.8%, respectively, *P* > 0.05; *n* = 5–9; Fig. 8 compared to Fig. 1).

## 12 Phosphorylation of β3 subunits alone does not account for protein kinase modulation of THDOC potentiation at α4β3δ GABA_A_ receptors

**Fig. 9:**
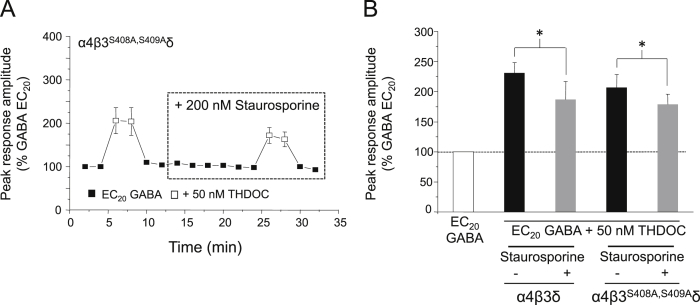
Staurosporine reduces THDOC potentiation at α4β3^S408A,S409A^δ GABA_A_ receptors. (A) Mean peak currents for α4β3^S408A,S409A^δ GABA_A_ receptors in response to EC_20_ GABA or EC_20_ GABA + 50 nM THDOC. Cells were treated with 200 nM staurosporine. (B) Bar chart showing the potentiation of EC_20_ GABA-activated currents by 50 nM THDOC before (black bars) and after (grey bars) staurosporine treatment in cells expressing α4β3δ (*n* = 9) or α4β3^S408A,S409A^δ (*n* = 7) GABA_A_ receptors. Data for α4β3δ receptors is taken from Fig. 8. *P < 0.05, paired *t*-test.

For synaptic-type α1β3γ2L GABA_A_ receptors, the extent of β3 subunit phosphorylation regulates the magnitude of neurosteroid potentiation (Fig. 5, Fig. 6). Equally, phosphorylation at the β3 subunit may also be important for the modulation of neurosteroid activity at extrasynaptic-type α4β3δ receptors. To this end we expressed GABA_A_ receptors containing mutant β3^S408A,S409A^ subunits, in combination with wild-type α4 and δ. Surprisingly, following staurosporine treatment, α4β3^S408A,S409A^δ GABA_A_ receptors still exhibited a significant reduction in THDOC-mediated potentiation (27.2 ± 4.6%, *P* < 0.05, paired *t*-test; *n* = 9; Fig. 9), comparable to that seen with α4β3δ receptors, an effect which was not observed in the equivalent synaptic-like receptor (Fig. 5C). Therefore it appears that phosphorylation at the β3 subunit is not the sole determinant of neurosteroid potentiation by protein kinases in α4β3δ receptors. However, staurosporine was notably less effective at α4β3^S408A,S409A^δ GABA_A_ receptors compared to α1β3γ2L single mutants (Fig. 5C), with a smaller decrease in potentiation being observed at these compared to wild-type receptors (27.2 ± 4.6% versus 38.1 ± 5.8%; Fig. 9B). This suggests that phosphorylation at the β3 subunit may play a role in the modulation of neurosteroid activity at α4β3δ receptors, but that phosphorylation at this subunit is insufficient to completely account for the modulatory effects of protein kinases observed at wild-type extrasynaptic-type receptors. Therefore, phosphorylation at other residues and/or subunits may also be important for the regulation of neurosteroid potentiation at this receptor subtype.

## 13 Phosphorylation of α4 and β3 subunits regulate neurosteroid activity at α4β3δ GABA_A_ receptors

**Fig. 10:**
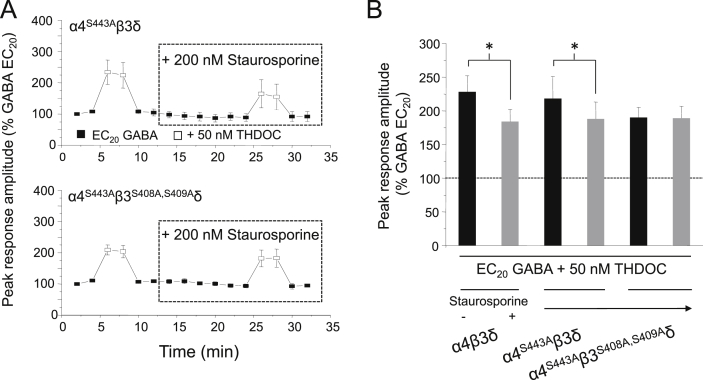
Staurosporine reduces THDOC potentiation at α4^S443A^β3δ, but not at α4^S443A^β3^S408A,S409A^δ GABA_A_ receptors. (A) Mean peak currents recorded from HEK293 cells expressing α4^S443A^β3δ (top panel: *n* = 7) or α4^S443A^β3^S408A,S409A^δ (bottom panel: *n* = 7) GABA_A_ receptors in response to EC_20_ GABA or EC_20_ GABA + 50 nM THDOC. Cells were treated with 200 nM staurosporine. (B) Bar chart showing the mean potentiation of EC_20_ GABA-activated currents by 50 nM THDOC before (black bars) and after (grey bars) staurosporine treatment in cells expressing α4β3δ (*n* = 9), α4^S443A^β3δ (*n* = 7) or α4^S443A^β3^S408A,S409A^δ (*n* = 7) GABA_A_ receptors. Data for α4β3δ receptors is taken from Fig. 8. *P < 0.05, paired *t*-test.

Another PKC phosphorylation site is known to exist on the α4 subunit, at serine 443 (Abramian et al., 2010), which is absent from other members of the α subunit family (Moss et al., 1992, Moss and Smart, 1996). This residue has recently been found to be responsible for the increased expression of α4 subunit-containing extrasynaptic receptors following exposure to neurosteroid (Abramian et al., 2014). To explore the involvement of α4^S443^ in the modulation of neurosteroid potentiation in our study, we used an S443A mutation to prevent phosphorylation at this site. Similar to the results obtained for α4β3^S408A,S409A^δ GABA_A_ receptors, cells expressing α4^S443A^β3δ exhibited a significant decrease in THDOC-mediated potentiation following treatment with 200 nM staurosporine (for 14 min: 24.5 ± 4.3%, *P* < 0.05, paired *t*-test; *n* = 7; Fig. 10). By contrast, for α4^S443A^β3^S408A,S409A^δ receptors, staurosporine was now ineffective as the magnitude of potentiation elicited by 50 nM THDOC was unaffected by staurosporine treatment (0.9 ± 6.5%, *P* > 0.05; *n* = 7; Fig. 10). Thus, phosphorylation at both the α4 and β3 subunits must be abolished in order to fully prevent the staurosporine-induced decrease in THDOC potentiation. This suggests that phosphorylation at both α4 and β3 subunits is important for regulating neurosteroid activity at α4β3δ GABA_A_ receptors. However, as neurosteroid potentiation was still evident at α4^S443A^β3^S408A,S409A^δ GABA_A_ receptors, (50 nM THDOC, 89.7 ± 15.3% enhancement of the EC_20_ GABA-activated current: Fig. 10), this confirms that, as for the synaptic GABA_A_ receptor counterpart, phosphorylation by protein kinases is not absolutely required to induce THDOC-mediated potentiation at extrasynaptic-like α4β3δ GABA_A_ receptors.

## 14 Discussion

Neurosteroids are potent modulators at the GABA_A_ receptor, potentiating GABA-mediated responses at low concentrations, and thereby enhancing levels of inhibition, but also directly activating the receptor at higher, often non-physiological concentrations (Belelli and Lambert, 2005). Specific residues that are common to all GABA_A_ receptor α subunits are responsible for these potentiating actions (Hosie et al., 2006, Hosie et al., 2009). As endogenous modulators of GABA_A_ receptor function, neurosteroids play an important role in mediating the stress response, and inducing anxiolysis or sedation, a phenotype that may feature during pregnancy or the ovarian cycle, when neurosteroid levels increase manifold (Bäckström et al., 2003, Belelli and Lambert, 2005, Purdy et al., 1991, Maguire et al., 2009, Maguire and Mody, 2007). This in turn can induce cyclic changes in δ subunit expression and a concurrent modulation of tonic inhibition in specific brain regions such as the dentate gyrus (Maguire and Mody, 2007). Protein kinases are also potent modulators of GABA_A_ receptor function in a bi-directional manner, achieved by targeting specific residues for phosphorylation. One of the more significant modulators is PKC which targets known serine residues to modulate GABA-mediated responses (Moss and Smart, 1996, Krishek et al., 1994, Lin et al., 1996, Leidenheimer and Chapell, 1997, Jovanovic et al., 2004, Brandon et al., 2000). Given that these endogenous modulatory mechanisms operate ubiquitously within and around CNS neurons, it is very likely that they will interact and may be synergistic in their modulation of GABA_A_ receptor function, especially during times of stress when neurosteroid levels are elevated.

There are many precedents for a positive synergistic interaction of the naturally-occurring neurosteroid, THDOC, and protein kinases (Leidenheimer and Chapell, 1997, Fáncsik et al., 2000, Harney et al., 2003), though equally there is evidence to the contrary (Koksma et al., 2003, Vergnano et al., 2007, Kia et al., 2011). This inconsistency may reflect the responses of different receptor subtypes to both neurosteroids and protein kinases (Belelli et al., 2002, Herd et al., 2007, Moss and Smart, 1996). With this in mind we have determined how both typical synaptic- (α1β3γ2L) and extrasynaptic-type (α4β3δ) receptor modulation by neurosteroid is influenced by the activity of protein kinases typical of neuronal cells.

We noted in this study that both synaptic and extrasynaptic recombinant receptor isoforms were equally sensitive to neurosteroid, with EC_20_ concentrations of GABA potentiated by ∼100% with 50 nM THDOC, underlying the consistency of the THDOC effect among receptor isoforms. Equally, the potentiated responses of both receptor types were sensitive to the broad-spectrum kinase inhibitor staurosporine, which typically inhibited by up to 50%. This demonstrates a consistent inhibition of the potentiating neurosteroid action by protein kinases, and also reinforces the instrumental role basal protein kinase activity plays in controlling the ability of neurosteroids to potentiate at these receptor isoforms. The similar level of potentiation observed with both types of GABA_A_ receptor is not surprising given that the neurosteroid binding site is conserved on all α subunits (Hosie et al., 2009) and these are common to extrasynaptic and synaptic receptors. Nevertheless, it is plausible that the neurosteroid allosteric modulation can be affected by other subunits in the pentamer. However, the sensitivity of extrasynaptic GABA_A_ receptors to neurosteroids, many of which will contain δ subunits, displays variability, and the underlying conditions causing this variation are yet to be established (Wang, 2011, Wohlfarth et al., 2002).

By searching for a mechanism, using specific kinase inhibitors and activators, we establish that PKC is largely responsible for these actions on synaptic receptors. Importantly, the activity of PKC is able to modulate THDOC potentiation over a broad spectrum of physiological concentrations of this neurosteroid. Candidate residues for phosphorylation by PKC have previously been identified as neighbouring serines 408 & 409 on the β3 subunit (Moss et al., 1992, McDonald and Moss, 1997). Mutating these residues individually in the synaptic-type receptor was insufficient to ablate the specific effects of PKC modulation, but substitution of both serines (β3^S408A,S409A^) prevented any modulation of THDOC potentiation by the both PMA and BIM-I. Clearly, both these residues are targeted by PKC for phosphorylation under both basal and enhanced phosphorylation conditions, and in order to establish the enhanced potentiation observed with 50 nM THDOC. The specificity of this synergistic interaction for this synaptic receptor was confirmed by Western blotting following substitution of S408 and S409, and the potentiating neurosteroid binding site, α1^Q241^. Clearly, the phosphorylation status of the synaptic receptor (α1β3γ2) is very important in revealing the full effect of THDOC, as increased phosphorylation enhances the actions of THDOC, whereas inhibition of phosphorylation impairs it.

Equally, our studies using staurosporine also revealed the same synergistic mechanism at work for extrasynaptic-like GABA_A_ receptors (α4β3δ). However, for this isoform, modulation of THDOC potentiation by protein kinase not only occurs via phosphorylation of S408 and S409 on the β3 subunit, but also occurs simultaneously via S443 on the α4 subunit, as only on the triple mutant receptor (α4^S443A^β3^S408A,S409A^δ) is staurosporine ineffective. In fact, the α4^S443^ site is exclusively phosphorylated by PKC, rather than by other kinases (Abramian et al., 2010). It is of interest to note that α1 and α4 subunits can also assemble with β2 subunits (Sieghart and Sperk, 2002, Olsen and Sieghart, 2009) and the latter are also substrates for PKC phosphorylation at S410 (Moss and Smart, 1996), which may also influence the extent of neurosteroid potentiation.

Collectively, our data suggest that THDOC can enhance GABA-activated currents at both synaptic and extrasynaptic receptors in a phosphorylation-independent manner, since by inhibiting PKC activity THDOC potentiation is reduced, though never abolished. However, as well as being inhibited, THDOC potentiation can also be augmented by the phosphorylation state of these receptors through the specific activation of PKC. Notably, β3^S408,S409^ and α4^S443^ are specifically targeted by PKC for phosphorylation (Abramian et al., 2010), and indeed, it has recently been suggested that THDOC may promote the activity or recruitment of PKC isoforms associated with the receptor, especially α4 subunit-containing receptors, in order to assist receptor phosphorylation (Abramian et al., 2014). We clearly show that residues β3^S408,S409^ are both targets for PKC modulation of the THDOC potentiation, though additionally α4^S443^ is also key in α4 subunit-containing extrasynaptic receptors. Current evidence suggests that only the α4^S443^ residue of extrasynaptic receptors is targeted for THDOC-induced phosphorylation by PKC to increase cell surface GABA_A_ receptor expression/stability, to bring about enhanced tonic (THIP-activated) currents in hippocampal slices (Abramian et al., 2014). The contribution made by β3^S408,S409^ to tonic neuronal currents is yet to be assessed, though, surprisingly, α1β3 cell surface levels were unaffected by THDOC. In our current study, total expression levels of β3 subunits in HEK cells, as part of the α4β3δ hetero-pentameric receptor, were unchanged after THDOC.

In conclusion, we find that phosphorylation of residues β3^S408,S409^ in addition to α4^S443^ in extrasynaptic receptors is a prerequisite for full THDOC-induced potentiation of GABA responses, and this must involve a signal transduction pathway linking the first α-helical transmembrane domain (M1) in the α subunits (location of a neurosteroid binding site) with the large intracellular region between M3 and M4 (sites for phosphorylation). We may speculate that although the M3-M4 loop lacks a defining physical structure to date, the interaction between neurosteroids and protein kinases may suggest this domain has a much closer association with the membrane based M1-M4. It also argues that other interacting molecules with the M3-M4 loop (receptor-associated proteins) and post-translational modifications (e.g., ubiquitination) may also have as yet undisclosed effects on neurosteroid potentiation.

## Author information

The authors declare no competing financial interests. Correspondence and request for materials should be addressed to TGS (t.smart@ucl.ac.uk).
